# Integration of a multi-omics stem cell differentiation dataset using a dynamical model

**DOI:** 10.1371/journal.pgen.1010744

**Published:** 2023-05-11

**Authors:** Patrick R. van den Berg, Noémie M. L. P. Bérenger-Currias, Bogdan Budnik, Nikolai Slavov, Stefan Semrau

**Affiliations:** 1 Leiden Institute of Physics, Leiden University, Leiden, Zuid-Holland, The Netherlands; 2 Mass Spectrometry and Proteomics Resource Laboratory, Harvard University, Cambridge, Massachusetts, United States of America; 3 Department of Bioengineering, Northeastern University, Boston, Massachusetts, United States of America; Max-Planck-Institut fur molekulare Physiologie, GERMANY

## Abstract

Stem cell differentiation is a highly dynamic process involving pervasive changes in gene expression. The large majority of existing studies has characterized differentiation at the level of individual molecular profiles, such as the transcriptome or the proteome. To obtain a more comprehensive view, we measured protein, mRNA and microRNA abundance during retinoic acid-driven differentiation of mouse embryonic stem cells. We found that mRNA and protein abundance are typically only weakly correlated across time. To understand this finding, we developed a hierarchical dynamical model that allowed us to integrate all data sets. This model was able to explain mRNA-protein discordance for most genes and identified instances of potential microRNA-mediated regulation. Overexpression or depletion of microRNAs identified by the model, followed by RNA sequencing and protein quantification, were used to follow up on the predictions of the model. Overall, our study shows how multi-omics integration by a dynamical model could be used to nominate candidate regulators.

## Author summary

Pluripotent stem cells, which can be derived from an adult individual, can be grown indefinitely in a dish and turned into each cell type of the body. These abilities enable applications of stem cells in basic research and regenerative medicine. Differentiation, the conversion into a precisely defined cell type, typically requires complex protocols that often have low efficiency. A better understanding of the molecular mechanisms underlying differentiation could help us improve existing protocols. Here, we studied the differentiation of embryonic stem cells induced by a small molecule (retinoic acid). We measured the abundances of three important classes of biomolecules–micro RNAs, messenger RNAs and proteins–at multiple time points during a 96 h-long differentiation experiment. We observed changes in the abundances of thousands of molecules. To make sense of these measurements we developed a mathematical model that connects the different classes of biomolecules and aims to predict their dynamics. Such models might help us identify new opportuntities to control differentiation at the molecular level. The data set we created, which we provide through an easily accessible web application, will also be a useful resource for other researchers interested in stem cell biology.

## Introduction

Much of the medical potential of pluripotent stem cells is due to their ability to differentiate into all cell types of the adult body [1]. While tremendous progress has been made in guiding cells through successive lineage decisions, the regulatory mechanisms underlying these decisions often remain unknown, especially at the post-transcriptional level. This gap in knowledge hampers the streamlining and acceleration of differentiation protocols.

A common first step towards finding novel gene regulatory relationships is the comprehensive, ideally genome-wide, measurement of gene expression dynamics. A large body of work has focused on charting transcriptome changes during differentiation, most recently down to the single-cell level [2–6]. While highly informative, such studies usually make the implicit assumption that mRNA levels are a good proxy for protein levels, despite widespread discordance observed in several mammalian systems [7–9]. The relationship between mRNA and protein abundance has been studied in various systems at different resolutions and time scales. Originally, mRNA-protein correlation was assessed across the genome (“across-gene correlation”[10]) in cell lines growing in steady state, where population-average abundances were measured with bulk omics methods. In this context, initial estimates claimed that only 40% of protein variability across the genome is explained by mRNA abundance in steady state [11]. Models of the protein to mRNA ratio explained up to two-thirds of the variability, when sequence features—such as the length of the coding sequence or amino acid frequencies—were taken into account [12]. Importantly, discordance between mRNA and protein abundance does not immediately imply specific regulation, as technical noise tends to reduce the observed correlation and conventional correction schemes typically ignore the effect of systematic, correlated errors [13]. In a comparison of relative protein levels across human tissues, about 50% of the variance in protein abundance was ascribed to post-transcriptional regulation [14]. All in all, a significant amount of protein abundance variability across the genome seems to remain unexplained, even if the effect of technical noise is considered.

Bulk measurements in unperturbed, steady state conditions can only reveal across-gene correlations. Single-cell methods or bulk measurements of dynamic systems, on the other hand, reveal fluctuations across cells or time, respectively, which allows us to study mRNA-protein correlation of individual genes (“within-gene correlation”[10]). The variability of mRNA-protein ratios in single-cells was found to be influenced by various factors such as protein half-life [15], as well as phenotypic state or microenvironment [16]. Within-gene mRNA-protein correlation can also be studied by measuring mRNA and protein abundances at the population level across time in a highly dynamic system, such as differentiating stem cells.

For this study, we collected a bulk multi-omics data set of retinoic acid (RA)-driven differentiation of mouse embryonic stem cells (mESCs). Samples taken over a period of 96 h were subjected to: mass spectrometry, bulk RNA-sequencing of nuclear and cytoplasmic fractions, as well as small RNA sequencing to quantify microRNA (miR) abundance. To describe protein dynamics, we refined a birth-death model [17–22] by considering explicitly the cytoplasmic fraction of the mRNA and the influence of certain technical artefacts related to mass spectrometry. In contrast to steady-state models, dynamical models aim to infer kinetic rates for protein synthesis and degradation rather than explain absolute protein levels. Here, we show how such models can in principle be used to nominate candidate regulators of gene expression during stem cell differentiation. By assuming a specific effect of miRs on protein synthesis, we attempted to identify miRs with a potential regulatory function. Finally, we used mimics and inhibitors of several candidate miRs to follow up on the model’s predictions.

## Results

### Pervasive discordance between mRNA and protein in retinoic acid-driven mESC differentiation

**Fig 1:**
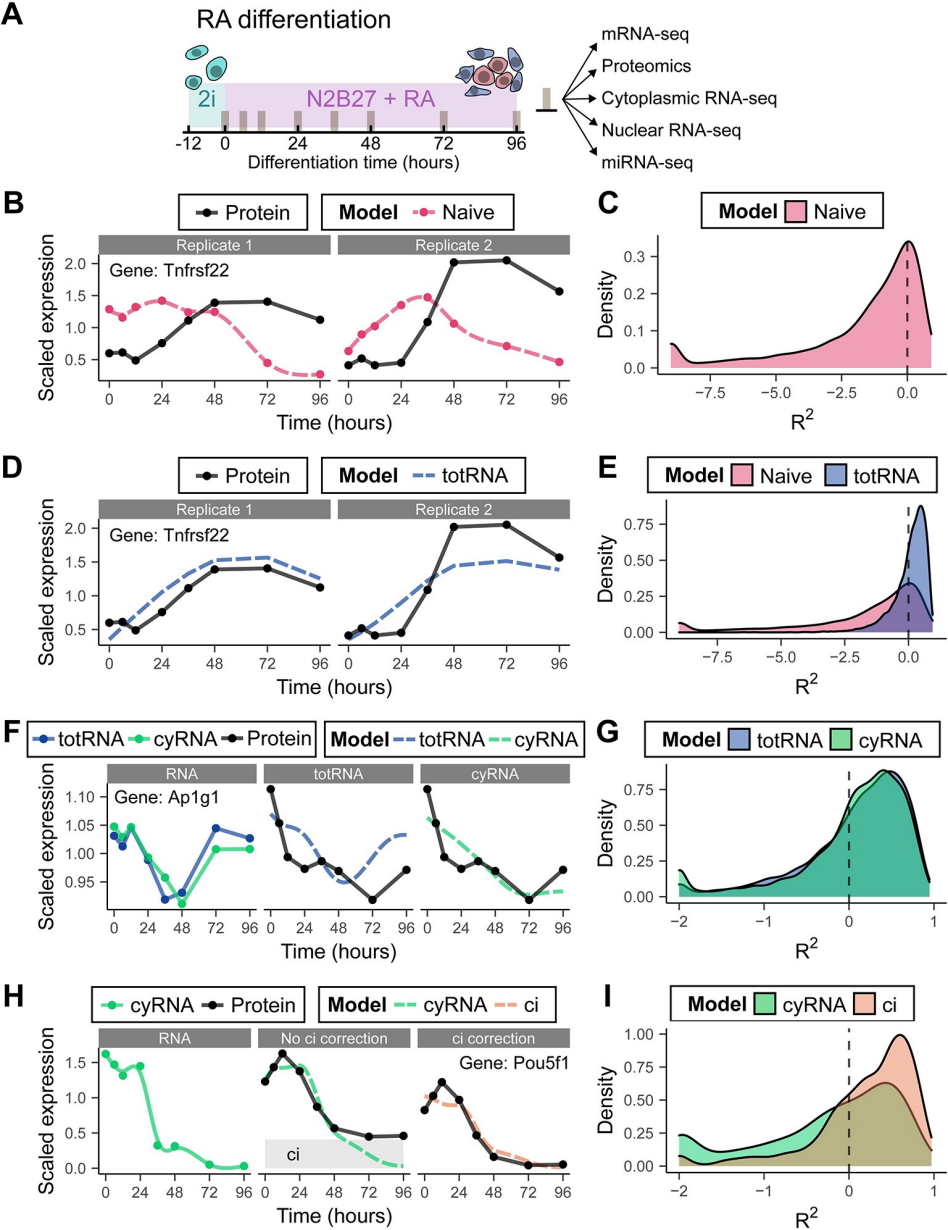
Birth-death models outperform the naive model in predicting dynamics. (A) Schematic overview of RA differentiation time course and subsequent omics measurements. (B) Example fit of the naive model. The *naive* model is a smoothing spline fit of RNA scaled to match the mean protein expression. (C) R^2^ distribution of the *naive* model. (D) Example fit of the *total RNA* (totRNA) model. (E) R^2^ distributions of the *naïve* and *total RNA* totRNA models. (F) Example fit of the *total RNA* and *cytoplasmic RNA* model, replicate 1. (G) R^2^ distributions of the *total RNA* and *cytoplasmic RNA* model. (H) Example fit of the *cytoplasmic RNA* and *ci* model, replicate 1. The height of the grey bar indicates the fitted *ci* parameter. (I) R^2^ distributions of the *cytoplasmic RNA* and *ci* model. Only genes that are improved by the *ci* model are shown. The distribution of all genes is shown in S2E Fig. (C,E,G,I) Some genes with extremely low R^2^ values are set to the minimum value of the plot for clarity. Corresponding Pearson’s r distributions are plotted in S2A–S2D Fig.

We used RA differentiation of mESCs as a generic model for *in vitro* differentiation. Previously, we characterized this differentiation assay in detail at the transcriptional level by single-cell RNA-seq [2] and showed that RA exposure induces a bifurcation into extraembryonic endoderm-like and ectoderm-like cells. Here, we collected RNA and protein samples during an RA differentiation time course (Fig 1A). For each time point, we quantified poly(A) RNA by RNA-seq and protein expression by tandem mass tag (TMT) labeling followed by tandem mass spectrometry (MS/MS). In total, we obtained RNA and protein abundance estimates for 6271 genes (S1A–S1E Fig) at 8 time points in duplicate. After correction for batch effects due to separate sequencing runs (S1F Fig), we achieved highly similar results for the two biological replicates. To investigate in how far protein expression can be predicted from RNA expression, we started with the simplest conceivable model (termed *naive* here), which assumes that protein abundance is connected to RNA expression by a constant, gene-dependent scaling factor. This model is justified, if protein synthesis and degradation rates are constant and RNA expression changes slowly on the time scale of protein turnover, thus resulting in a quasi-steady state. Consequently, the protein-to-RNA ratio would be approximately constant over time. To test this model, we scaled both protein and RNA to their respective means, which should result in a constant protein-to-RNA ratio of 1, if the *naive* model is valid. We observed that for a large fraction of genes, the *naive* model is inaccurate, resulting in low coefficients of determination (R^2^) and low correlation (Figs 1B, 1C and S2A). In many cases, R^2^ assumes negative values, which means that the *naive* model performs worse than a model predicting a scaled protein level of 1 for all time points. (Note that only for linear regression models R^2^ is guaranteed to be positive and interpretable as the fraction of variance explained by the model.) For some genes, we even observed significant anti-correlation between RNA and protein (Fig 1B). While technical noise certainly contributes to this result, the high quality of our data sets (S1 Fig) suggests that a substantial part of the observed discordance is of biological origin. The assumptions of the *naive* model are therefore likely wrong for the majority of genes and a more sophisticated model is necessary to explain the relationship between RNA and protein.

### A simple birth-death model explains mRNA-protein discordance for most genes

To relax the assumption that expression is in steady state, we next considered a kinetic model that implements a birth-death process for protein dynamics (Eq 1).


$${\dot{P}}^{g}(t)=k_{s}^{g}\cdot R^{g}(t)-k_{d}^{g}\cdot P^{g}(t)\;{\mathrm{with}\;k}_{s}^{g}>0,k_{d}^{g}>0$$ (1)

In this *total RNA* model, protein production of gene *g* depends on total mRNA abundance *R* and the protein synthesis rate *k*_*s*_, while protein degradation depends on protein abundance *P* and the degradation rate *k*_*d*_. Synthesis and degradation rate are taken to be constant in time but gene-specific (as indicated by the index *g*). All processes related to protein production (initiation, elongation, etc.) are lumped into k_s_, while k_d_ represents all processes leading to a reduction in protein levels (dilution due to cell division, active degradation, etc.). Similar models have been used previously to describe protein dynamics during the stress response in yeast [18], as well as embryonic development of *Xenopus* [19] and *Drosophila* [22]. We do not consider simpler, degenerate models (without k_s_ and/or k_d_ [19]), because these models are not relevant in our biological system: Synthesis and degradation always occur to some degree during stem cell differentiation. To reduce the influence of uninformative small fluctuations, we applied a smoothing spline to the abundance estimates prior to inferring model parameters by non-linear least-squares fitting. Compared to the *naive* model, R^2^ and correlation improved markedly for the *total RNA* model (Figs 1D, 1E and S2B), which is to be expected given the increase in model flexibility. To correct for a difference in the number of fit parameters and thus compare model performance fairly, we used the Bayesian information criterion (BIC, see Methods). According to the BIC, 3551 out of 4580 proteins were better fit by the kinetic model. These proteins are thus likely out of steady state, for the duration of the experiment, as a result of the differentiation cue. In summary, these results showed that a simple birth-death model outperforms the *naive* model of protein dynamics.

Despite the overall improvement observed with the *total RNA* model, R^2^ and correlation were still low for many proteins. We hypothesized that the remaining discrepancies could be explained by kinetic rates changing over time. To evaluate this hypothesis, we first sought to exclude technical limitations of our measurements as possible alternative explanations. We first considered the subcellular localization of mRNA. In our first experiment, we measured total poly(A) RNA, whereas only cytoplasmic mRNA is available for translation. Nuclear retention of mRNA is known to reduce transcriptional noise [23] and has been shown to contribute to translational regulation for specific genes [24–26]. To measure the cytoplasmic mRNA fraction of each gene, we repeated the differentiation experiment in triplicate and separated cell lysates into a nuclear and a cytoplasmic fraction before performing RNA-seq. To obtain a global scaling factor between cytoplasmic and nuclear expression, we regressed total RNA (totRNA) reads, measured previously, on nuclear RNA (nuRNA) reads and cytoplasmic RNA (cyRNA) reads across all genes (see Methods). Then, the cytoplasmic fraction *C* was calculated for each gene and each time point. To our surprise, *C* did not vary substantially between genes (mean = 0.82, std = 0.02, calculated for a subset of 3,563 genes without any missing values) (S1G Fig). In addition, *C* also did not fluctuate much in time for individual genes (S1H Fig). Despite the low variability of *C*, we incorporated this parameter, leading to the *cytoplasmic RNA* model (Eq 2).


$${\dot{P}}^{g}(t)=k_{s}^{g}\cdot C^{g}(t)\cdot R^{g}(t)-k_{d}^{g}\cdot P^{g}(t)$$

$$\mathrm{with}\;0\leq C^{g}(t)\leq 1$$ (2)

As to be expected, adding *C* brought overall only a subtle improvement (Fig 1G), although for individual cases, the improvement was significant (Fig 1F). We opted to fit further models including the cytoplasmic fraction, due to the overall slightly better performance.

Another potential confounder is inherent to the proteomics method we employed. TMT-based proteomics suffers from co-isolation interference (ci), a process in which two peptides are co-isolated in the second MS step. The contaminating peptide can interfere with the quantification of the peptide of interest and cause a constant offset [27,28]. To model a possible offset, we extended the model by an additional parameter (*ci*), quantifying the degree of fold-change compression for a protein. Thus, we assume *ci* to be constant for all TMT tags, i.e. time points, resulting in the *ci* model (Eq 3).


$${\dot{P}}^{g}(t)+ci^{g}=k_{s}^{g}\cdot C^{g}(t)\cdot R^{g}(t)-k_{d}^{g}\cdot (P^{g}(t)+ci^{g})$$

$$0\leq ci^{g}\leq min\{P^{g}(t)\}$$ (3)

Effectively, including the parameter *ci* allows protein expression to have a bigger dynamic range, which can improve the fit for certain genes significantly (Figs 1H, 1I, S2D and S2E). Judged by the BIC, 557 genes were fit better including *ci*. All in all, this result reinforces the importance of considering co-isolation interference.

### Including miRs improves model performance and identifies miR-mRNA interactions

Having incorporated important confounding factors, we sought to further extend the model by relieving the assumption of constant kinetic rates. Such a model would be able to capture protein synthesis and degradation varying across time, which likely happens during a process as dynamic as stem cell differentiation. A model in which *k*_*s*_ and *k*_*d*_ are time-dependent and completely arbitrary cannot be sufficiently constrained by our mRNA and protein measurements. Hence, we decided to focus on a specific regulatory mechanism, for which an additional, complementary data set could be obtained. Specifically, we explored the influence of miRs, which are known to be involved in gene regulation during differentiation [29]. In order to study the role of miRs in our system, we repeated the RA differentiation assay and measured the miRnome by small RNA-seq in quadruplicate. We quantified around 1000 mature miRs per time point (S1A, S1C and S1E Fig). For further analysis, we retained miRs with high reproducibility across replicates and high variance across time (S1I Fig). To identify possible effects of miRs, we focused on computationally predicted miR target genes. We further reduced the number of miR-mRNA interactions by filtering based on the “context score” provided by TargetScanMouse [30] (S1D Fig). This score combines information from mutiple sequence features and can be used to rank hits. In the end we retained 4527 genes, 560 unique mature miRs and 45,882 potential interactions between them (S1D and S1E Fig).

**Fig 2:**
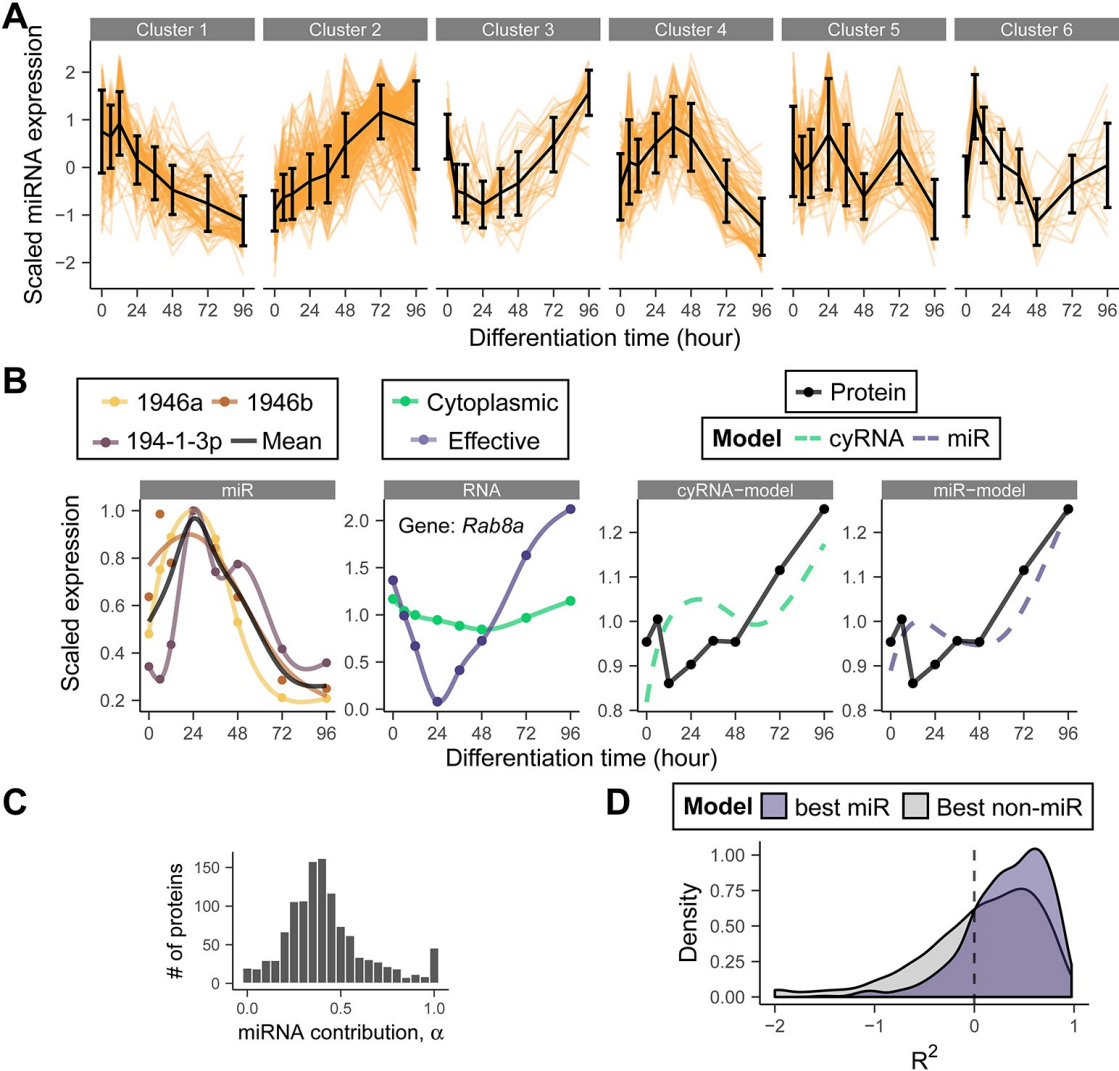
The addition of miRs further improves the dynamical model for a subset of genes and suggests potential miR-mRNA interactions. (A) Expression profiles of 560 miRs in six clusters. (B) Example fit of miR model for the gene *Rab8a*, replicate 1. First panel: expression of the assigned miRs of a single cluster. Colored lines are individual smoothing spline fits. Second panel: Cytoplasmic RNA expression and the effective RNA concentration available for translation (see Methods). Solid lines represent smoothing splines. Third/fourth panel: *cytoplasmic RNA* and *miR* model fits. (C) Distribution of inferred α for genes that benefit from *miR* model. (D) R^2^ distribution of the *miR* model and the next best model (either *naive*, *total RNA*, *cytoplasmic RNA* or *ci*). Only genes that benefit from the *miR* model are shown. Some genes with extremely low R^2^ values are set to the minimum value of the plot for clarity. The corresponding Pearson’s r distribution is shown in S2F Fig.

If multiple miRs with similar temporal profiles targeted the same gene, we considered them to be indistinguishable. Therefore, we grouped all miRs into six clusters by their temporal expression profiles (Fig 2A) and averaged over miRs from the same cluster targeting the same mRNA (Fig 2B). As miR binding is known to trigger translational repression [31], we modified the term describing protein synthesis in our model. In the interest of parsimony, we assumed a linear dependence of protein synthesis on miR abundance for this *miR* model (Eq 4).


$${\dot{P}}_{m}^{g}(t)=k_{s}^{g}\cdot (1-\alpha_{m}^{g}\cdot M_{m}^{g}(t))\cdot C^{g}(t)\cdot R^{g}(t)-k_{d}^{g}\cdot P^{g}(t)$$

$$0<\alpha_{m}^{g}\leq 1$$ (4)

Here, *M*^*g*^_*m*_ is the geometric mean abundance (scaled to its maximum across time) of miRs in cluster *m* targeting gene *g* and *α*^*g*^_*m*_ parametrizes the interaction between those miRs and the target gene *g*. For each gene, we fit a model with one of the six miR clusters at a time and identified the improvement in model performance. Including miRs greatly improved the fits for some proteins, especially when there was a transient discordance between RNA and protein expression (Fig 2B). Typically, the "effective" mRNA abundance (cytoplasmic mRNA corrected for miR effects) was more dynamic than nominal mRNA abundance. For many of the genes that benefited from the addition of miRs, their influence is typically large: For these genes, translation was reduced up to 50% by the miR term in the model at peak miR expression (Fig 2C). Overall, the addition of a miR-dependent term significantly improved the coefficient of determination for a quarter of the proteins as determined by the BIC (Fig 2D). At this point we would like to stress that we made particular assumptions about the influence of miRs (miR binding only affects k_s_ and the effect is linear in the abundance etc.). A more general model would have been under-constrained by the available data. Other conceivable interaction terms might result in improvements for different sets of genes. A better performance of the *miR* model, compared to simpler models, therefore does not prove the assumed regulatory mechanism.

### The best dynamical model explains 45% of total protein variance

**Fig 3:**
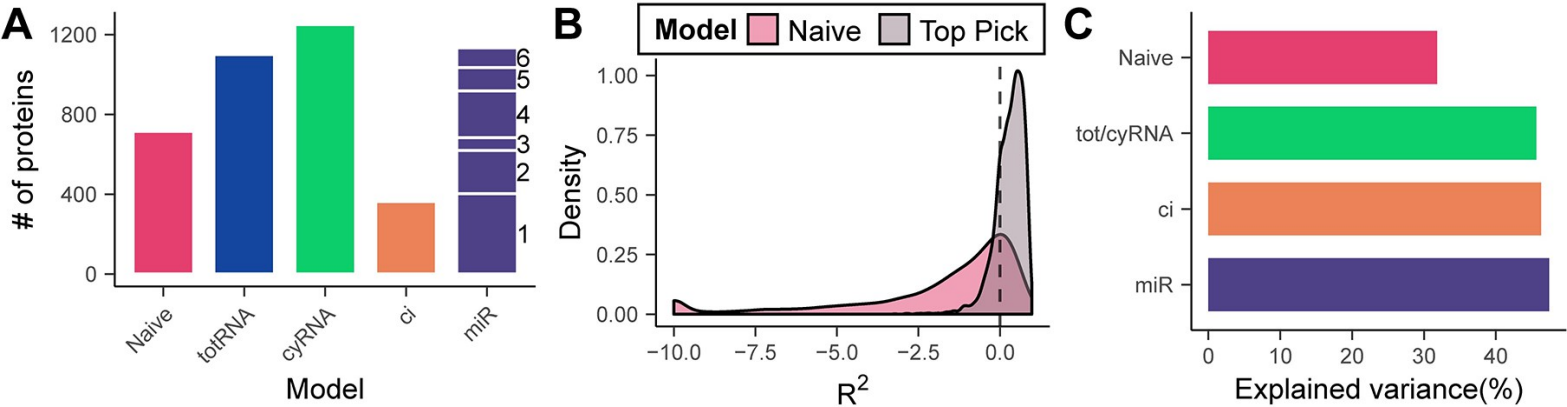
Selecting the optimal model on a gene-by-gene basis increases the total explained variance of protein expression from 30% to 50%. (A) Assignment of the optimal model for each gene based on the BIC. The number next to the miR bar indicates the miR cluster giving the best fit. (B) R^2^ distribution of the optimal fits from (A) and their naive model counterpart. Some genes with extremely low R^2^ values are set to the minimum value of the plot for clarity. (C) Median percentage of protein variance explained by each model. For each model, only those genes were included for which that model was the best. Fits with negative R^2^ were ignored.

While each model refinement introduced above improved model performance overall, each model achieved the lowest BIC only for a subset of genes (Fig 3A). In about 16% of cases the *naive* model was optimal, meaning that for these proteins none of the other models improved prediction by a significant amount (as judged by the BIC). 25% and 26% of genes were best predicted by the model without or with considering mRNA localization, respectively. Hence, for 51% of genes, protein abundance seemed to be out of steady state, but explainable by a simple model with fixed synthesis and degradation rates. For 8% of proteins, the model including co-isolation interference was optimal. The increased relative dynamic range due to subtracting a constant increased the fit for these genes significantly. Finally, 25% of proteins were fit optimally with a model including one of the miR clusters, indicating that there are likely additional regulatory mechanisms at play, potentially mediated by miRs. If the optimal model for each protein is chosen, only very few cases of negative R^2^ values remain (Fig 3B). Ignoring those, we found that the *miR* model explained 45% of the variance, when it is the optimal model (Fig 3C). In summary, the dynamic responses of 84% of the quantified proteins were signfificantly different from their mRNA counterparts. Therefore, it does not seem warranted to consider mRNA abundance a good proxy for protein levels in a highly dynamic setting.

### Follow-up on the model predictions using miR inhibitors and mimics

**Fig 4:**
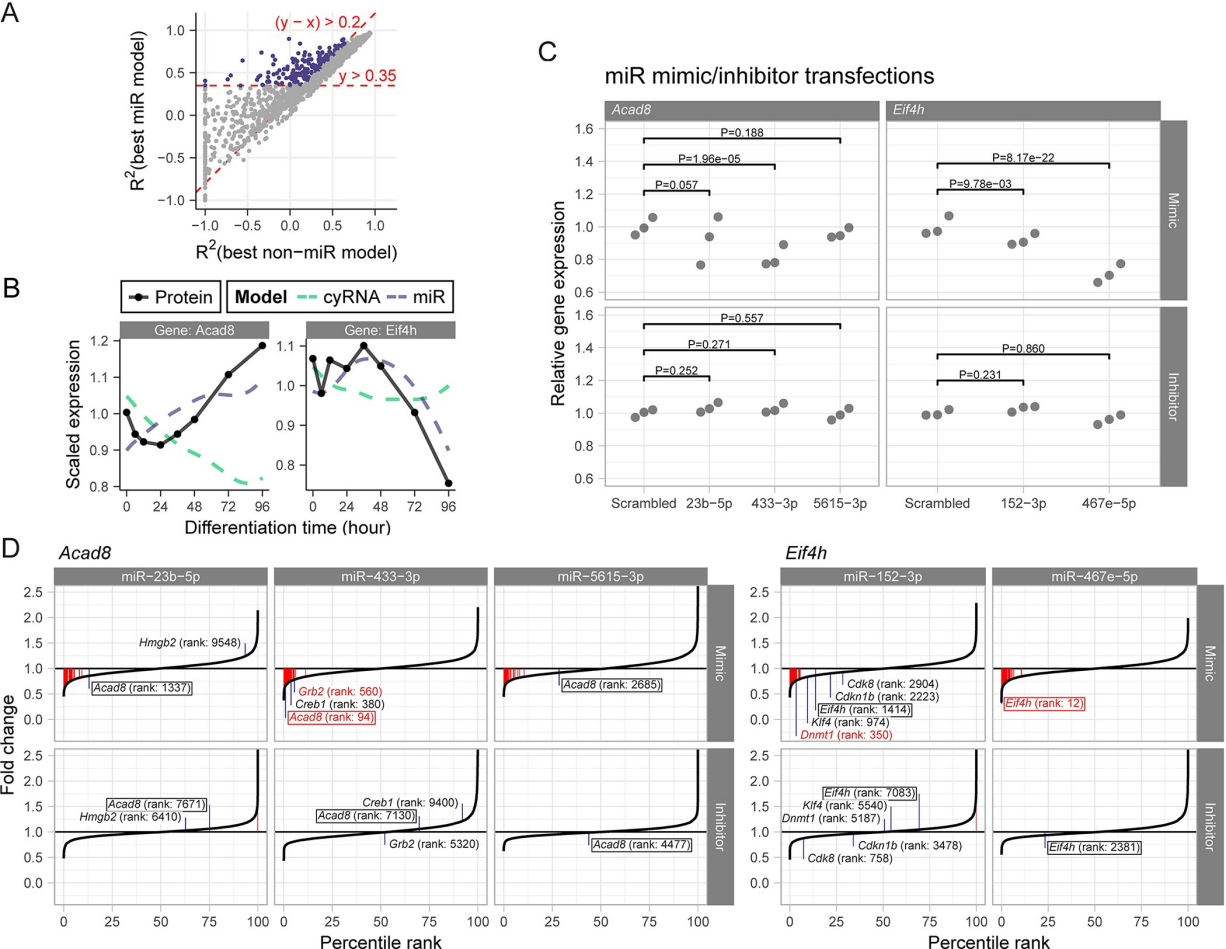
RNA-seq of mESCs transfected with mimics or inhibitors of miRs identified by the model. (A) Scatter plot of the R^2^ values for the best *miR* model and the best non-*miR* model, see Fig 2D. Colored dots are defined by the cutoffs indicated in red and represent a subset of genes with a miR-mRNA interaction of higher confidence. Some genes with extremely low R^2^ values are set to the minimum value of the plot for clarity. (B) miR model fits of *Acad8* and *Eif4H*, which belong to the subset highlighted in (A). (C) Expression levels (regularized counts scaled to scrambled control) of *Acad8* and *Eif4H* after miR mimic (top) and miR inhibitor (bottom) transfection in 3 biological replicates. P-value shown is for an uncorrected one-sided test (see Methods). Differential expression of six more target genes is shown in S5 Fig. (D) Expression fold changes relative to scrambled control after mimic and inhibitor transfections for three miRs that target *Acad8* and 2 miRs that target *Eif4h*. Distributions of the six more targets are shown in S6 Fig. The boxed genes are our proposed targets, additionally some known targets are shown. Red color indicates significantly differentially expressed genes (P_adj_ < = 0.01).

Given that the model including miRs was optimal for 25% of proteins, we wanted to explore in how far our model is able to identify candidates for novel miR-mRNA interactions. To that end, we ranked all proteins by model performance (R^2^) and performance improvement (i.e. change in R^2^) compared to the simpler models without miRs (Fig 4A and S1 Table). Among this list of candidate genes, we selected seven genes (*Rab8a*, *Cdk7*, *Pccb*, *Acad8*, *Mfge8*, *Eif4h* and *Srgap2*) and their thirteen putative regulating miRs (Figs 2B, 4B and S3), for further investigation. To test the functional significance of those miRs, we set up a transfection assay with miR mimics and inhibitors in mESCs. To optimize mimic and inhibitor transfection, we first created two fluorescent reporter cell lines, based on a published approach [32,33] (S4A Fig). In these cell lines, a bi-directional promoter drives the expression of two fluorescent proteins, functioning as miR ‘reporter’ and ‘normalizer’, respectively. The bi-directional promoter guarantees highly correlated levels of transcription of both transcripts. Due to miR binding sites in the ‘reporter’ transcript, its expression is reduced relative to the ‘normalizer’, if the respective miR is present. We created reporter cell lines for a miR that is undetected in our system (mir-590-3p) and one that is highly expressed (miR292a-5p), in order to evaluate a mimic and an inhibitor, respectively. Flow cytometry measurements of the mir-590-3p reporter line transfected with a mir-590-3p mimic revealed a high percentage of transfected and regulated cells after 24 h (S4B Fig). Although the effect increased slightly over time, we picked 24 h as the ideal time point for evaluation in order to limit the extent of secondary effects (S4C Fig). The miR292a-5p inhibitor was slightly less effective in modulating miR292a-5p reporter signal, even at higher doses (S4D Fig). For the miR inhibitor, we selected 48 h transfection with 2X the suggested concentration (S4E Fig).

Having set up optimal concentrations and timings for our transfection assays, we next set out to validate the predicted miR targets. Although our kinetic model was developed to predict regulation at the level of translation, we used mRNA abundance as a first, convenient readout, reasoning that mRNA degradation usually follows translational repression [31]. Cells were transfected with miR mimics and inhibitors for the thirteen aforementioned miRs and one additional miR as a control, for which we expected no effect (miR-216a-5p, which is predicted to target *Leo1*). mRNA samples from these experiments were subjected to RNA-seq. Differential expression analysis revealed significant downregulation in four out of eight targeted genes by at least one of the miR mimics (Figs 4C and S5A). *Acad8* was downregulated by miR-433-3p (P = 1.7e–3), *Cdk7* was downregulated by miR-99a-5p (P = 0.014), *Eif4h* was downregulated by both miR-152-3p (P = 0.015) and miR-467e-3p (P = 1.21e–19) and *Srgap2* by miR-135b-3p. The observed downregulation supported the hypothesis that miR-mRNA interaction takes place in these five particular cases. The miR inhibitors on the other hand did not result in significant differential expression in any of the predicted targets (Figs 4C and S5B Fig). The negative control *Leo1* was not differentially expressed, as expected (S5 Fig). Overall, the mimics of the thirteen miRs downregulated the predicted targets significantly in a combined analysis (P_fisher_ = 2.4e-3). On the contrary, the inhibitors did not have a significant opposite effect (P_fisher_ = 0.6), possibly due to a smaller change in miR abundance, compared to the mimics, and redundancy created by multiple miRs targeting the same gene.

As additional validation of our assay we looked at the measured differential expression for known targets of the perturbed miRs (Figs 4D and S6). Specifically, we focused on the miRs predicted to regulate *Acad8* and *Eif4h*, which gave the largest effect sizes at the mRNA level. miR-23b-5p, which we predicted to target *Acad8*, is also known to target *Hmgb2* [34] and thereby play a role in cardiac hypertrophy. Another predicted regulator of *Acad8* was miR-433-3p, which regulates genes involved in development, like the transcription factor *CREB* [35] and the *Egfr* binding adaptor protein *GRB2* [36]. The third predicted regulator of *Acad8*, miR-5615-3p, does not have any confirmed targets to the best of our knowledge. *Eif4h* was predicted to be regulated by miR-152-3p and miR-467e-3p the latter of which has no independently validated targets. Some of miR-152-3p’s known targets are the two cell cycle genes *CDKN1B* [37] and *CDK8* [38], the pluripotency inducing *KLF4* [39], and the DNA methyltransferase *Dnmt1* [40]. With the exception of *Hmgb2*, all known targets were downregulated in the respective mimic assays, indicating that our experiments can validate existing interactions.

**Fig 5:**
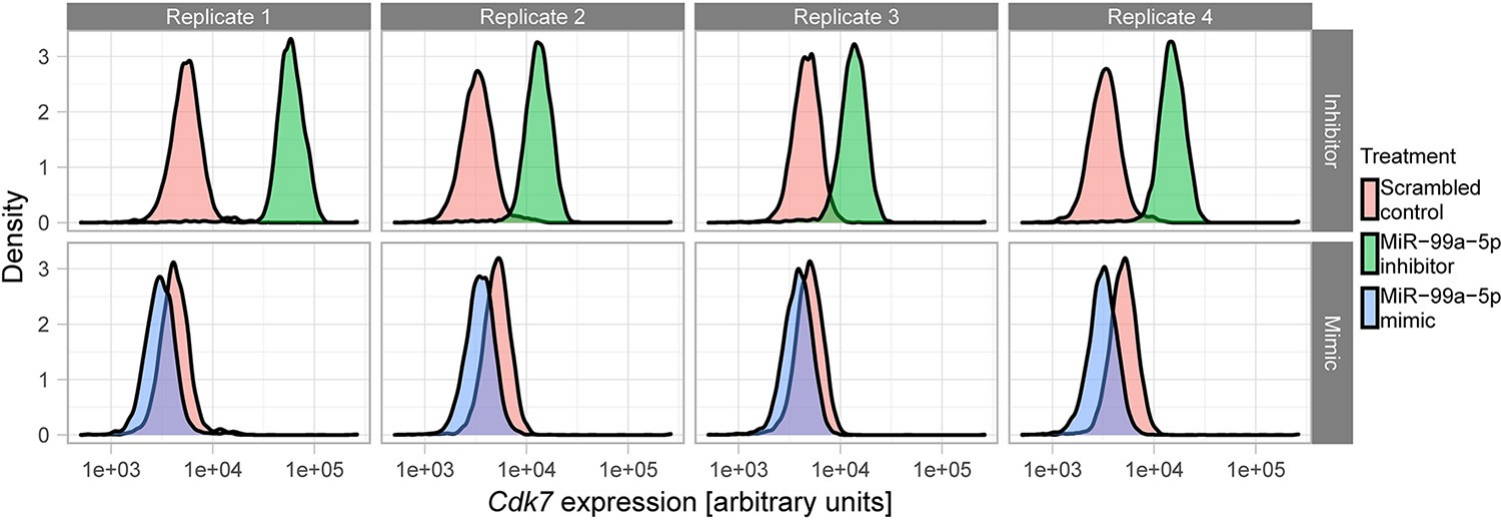
CDK7 protein abundance is regulated by miR-99a-5p in mESCs. Flow cytometry of CDK7 immunostaining in 4 biological replicates of mESCs treated with miR-99a-5p mimic, inhibitor or the respective scrambled controls.

Finally, we wanted to test, whether some of the identified miR-mRNA interactions also have an effect on protein abundance. We repeated the transfections with miR inhibitors and mimics for miR433-3p (which targets *Acad8*), miR467e-5p (which targets *Eif4h*) and miR-99a-5p (which targets *Cdk7*) in mESCs and measured protein abundance by immunofluorescence and flow cytometry. For these experiments we used a 5X reagent concentration to increase the expected effect size. In the case of ACAD8 and EIF4h, both miR mimic and inhibitor lead to effects in the same direction, relative to the respective scrambled control (S7 Fig). For ACAD8, both the mimic and the inhibitor caused an increase in protein abundance, while for EIF4H both treatments caused a decrease in abundance. This observation can be explained by other regulatory mechanisms that (over)compensate for the perturbations or secondary effects of the selected miRs. For CDK7, miR-99a-5p inhibition resulted in a strong upregulation of protein abundance, while the miR mimic slightly reduced abundance (Fig 5). This observation is consistent with miR-99a-5p specifically targetting CDK7. As shown above, miR-99a-5p inhibition did not significantly increase *Cdk7* mRNA levels (S5B Fig), which means that regulation occured at the level of translation.

All in all, the validation experiments showed that our model has the power to nominate potential regulators of protein abundance.

## Discussion

In this study we set out to integrate a multi-omics data set on stem cell differentiation. A range of tools for the integration of multiple omics modalities, both at the bulk and single-cell cell level [41,42], already exist. The most recent approaches have started to aim for biologically meaningful integration by incorporating prior knowledge in various ways. For example, biological knowledge can inform priors of Bayesian models or the topology of networks that represent interactions connecting different modalities [43,44]. A third way to exploit biological relationships between data sets, which we adopted in this study, is the use of dynamical models [17–22]. These models incorporate biophysical relationships between different types of molecules in a quantitative way and can also be used to infer kinetic parameters that have obvious biological interpretations^17–22^. We speculate that combinations of the mentioned approaches, exemplified by physics or systems-biology informed neural networks [45,46], will become powerful tools for data integration in the future.

In our time-resolved multi-omics data set we found overall low correlation between mRNA and protein abundance across time. Such low correlation has been observed in several systems, in particular: *Xenopus* development [19], *C*. *elegans* development [47], macrophage differentiation [48], mouse ESC differentiation [49] and the intestinal epithelium [50]. While the lack of strong correlation is typically interpreted as a sign of (post-)translational regulation [47,49], theoretical work showed that a simple delay between mRNA and protein production can lead to a reduction in gene-wise correlation [51,52]. A minimal model with constant kinetic rates explained the protein dynamics of a third of all genes during the stress response in yeast [18] and 75% of genes in *Xenopus* development [19]. In our system, 3552 out of 4580 genes were explained better by this model, compared to a naive model which assumes a constant protein-to-RNA ratio.

To explain the remaining discordance we explored the cytoplasmic fraction of the mRNA, but did not find a strong effect. A possible explanation could be that the nuclear fractionation method we used was not very effective and a substantial amount of cytoplasmic RNA remained in the nuclear fraction. While the mean cytoplasmic fraction measured here (0.82) was comparable to values reported in another study [23] of pancreatic beta cells (0.79) and liver cells (0.87), we observed much lower variability between genes compared to that previous study. This might either indicate that nuclear retention does not play a role in differentiation or that the nuclear fractionation method was not optimal. Intriguingly, Halpern et al. have shown, using single-cell methods and a dynamical model of mRNA turnover and nuclear export, that nuclear retention can reduce the variability of cytoplasmic mRNA abundance [23]. Reduced cytoplasmic noise likely leads to an increase in mRNA-protein correlation. It would be very insightful to employ single-cell methods and test whether similar mechanims are at play in stem cells and during differentiation. Whereas incorporating the cytoplasmic fraction only resulted in a small improvement, a model that includes co-isolation interference was optimal for almost 400 proteins. This result is in agreement with the known, confounding influence of co-isolation interference [27,28].

Overall, our analysis showed that for 16% of the proteins, the naive model is sufficient. From the mRNA abundances alone it is, however, impossible to predict, which genes would fall in this category. These results reinforce the notion that mRNA abundance should not be used without caution as a proxy for protein abundance [53]. Future work might reveal predictors (such as mRNA sequence composition, known binding motifs etc.) that might help to identify the regulatory mode of a particular gene. It will be interesting to see how much of the variance that remains unexplained by our most detailed model (55%) is due to technical noise or unexplored biological factors (regulation of protein degradation, control of protein turnover by RNA binding proteins etc.)

Having defined a simple birth-death model that includes the confounding effect of co-isolation interference, we next explored whether considering miRs could further improve model performance. miRs have been identified as key regulators of stem cell pluripotency and differentiation [29,54]. For example, members of the let-7 and miR-290 families have been implied as drivers of differentiation as well as the maintenance of pluripotency in ESCs [54–57]. To find putative targets of miRs, various computational methods, typically based on sequence complementarity and conservation, have been developed [30,58,59]. These methods predict hundreds of thousands of interactions, among which are likely many false positives. The gold standard for validation, the luciferase assay, is time-consuming, which means that the majority of potential interactions have not been verified. More comprehensive, experimental methods to identify miR-mRNA interactions, such as HITS-CLIP or PAR-CLIP employ crosslinking of the RNA-induced silencing complex (RISC) with associated miRs and their targets, but they can suffer from low sensitivity and require the use of reagents that might perturb cell physiology [60]. Our modeling-based approach might complement these methods in several ways: No special reagents are necessary and miR-mRNA interactions are identified by effect size, which might help to find functionally relevant miRs. Our approach identified several candidate regulators and we were able to validate the repression of CDK7 protein expression by miR-99a-5p. Future studies might reveal the functional relevance of this interaction for the maintenace of pluripotency, differentiation or other aspects of stem cell biology. Once more we would like to stress that the miR effect incorporated in our model is an assumption. Our model is consistent with the finding that miRs tend to repress translation, next to promoting mRNA degradation [61]. Yet, due to the under-constrained nature of the problem, a better fit of a model incorporating the assumed miR effect is not to be taken as evidence that this effect is (exclusively) at play. Our follow-up experiments suggested the presence of additional effects, not considered in our model, that significantly modulate the effect of miR inhibition or overexpression. To demonstrate the postulated regulatory mechanism conclusively, protein translation would have to be measured directly, for example by ribosome profiling or pulse-chase labeling and mass spectrometry [62].

In conclusion, our study demonstrates that biological relationships between datasets can be leveraged for the integration of multi-omics experiments. Developing optimal integration algorithms will be a continuing challenge as more and more types of molecular profiling data become available. We hope that our time-resolved multi-omic dataset will be a rich resource for the discovery of gene regulatory mechanism in stem cell differentiation.

## Materials and methods

### Experimental methods

#### Cell culture

E14 mouse embryonic stem cells were cultured as previously described [2]. Briefly, cells were grown in modified 2i medium [63]: DMEM/F12 (Life technologies) supplemented with 0.5x N2 supplement, 0.5x B27 supplement, 4mM L- glutamine (Gibco), 20 μg/ml human insulin (Sigma-Aldrich), 1x 100U/ml penicillin/streptomycin (Gibco), 1x MEM Non-Essential Amino Acids (Gibco), 7 μl 2-Mercaptoethanol (Sigma-Aldrich), 1 μM MEK inhibitor (PD0325901, Stemgent), 3 μM GSK3 inhibitor (CHIR99021, Stemgent), 1000 U/ml mouse LIF (ESGRO). Cells were passaged every other day with Accutase (Life technologies) and replated on gelatin coated tissue culture plates (Cellstar, Greiner bio-one). During transfections, cells were temporarily cultured in serum+LIF medium (10% ES certified FBS, 1X non-essential amino acids, 0.1mM β-mercaptoethanol, 1X pen/strep, 2mM L-glutamine, 10,000U/ml mLIF, mLIF from Merck, rest from Thermo Fisher Scientific). miR reporter cell line clone selection took place on homegrown mouse embryonic fibroblast feeders.

#### Retinoic acid differentiation and sample collection

Retinoic acid induced differentiation was carried out exactly as described before [Semrau:2016fu]. Prior to differentiation cells were grown in 2i medium for at least 2 passages. Cells were seeded at 2.5e5 per 10 cm dish and grown over night (12 h). Cells were then washed twice with PBS and differentiated in basal N2B27 medium (2i medium without the inhibitors, LIF and the additional insulin) supplemented with 0.25 μM all-trans retinoic acid (RA, Sigma-Aldrich). Spent medium was exchanged with fresh medium after 48 h. To collect samples, cells were dissociated with Accutase and spun down. Full RNA and cytoplastmic/nuclear RNA were always immediately extracted (RNeasy, Qiagen and SurePrep, Fisher Scientific, resp.) and the purified RNA was stored at -80C until RNA-sequencing was performed. For proteomics and miR-sequencing, pellets were flash frozen in liquid nitrogen and stored at -80C until further processing.

#### Cloning

The miReporter backbone (AddGene, Plasmid #82478) was transformed into DH5a competent cells (Cat. 18265017, Thermo Fisher Scientific) as per manufacturer’s instructions. Then, transformed cells were expanded and plasmids harvested by miniprep (Qiaprep, Qiagen). A set of two oligos was synthesized for each of the two reporter cell lines: miR-590-3p-fwd: 5’-GATCG TAATTTTATGTATAAGCTAGT AAGCTTC-3’, miR-590-3p-rev: 5’-CTAGGAAGCTT ACTAGCTTATACATAAAATTA C-3’, miR-292a-5p-fwd: 5’-GATCG ACTCAAACTGGGGGCTCTTTTG AAGCTTC-3’, miR-292a-5p-rev: 5’-CTAGGAAGCTT CAAAAGAGCCCCCAGTTTGAGT C-3’ (Integrated DNA Technologies, see S4A Fig). Pairs of oligos were annealed and phosphorylated in a thermocycler: 30m at 37°C, 5m at 95°C, for 12 cycles (1μM fwd oligo, 1μM rev oligo, 1X T4 buffer, 1U/μl T4 Polynucleotide Kinase; buffer and enzyme from New England Biolabs). Next, backbone digestion and ligation was performed in one step in a thermocycler, which was facilitated by the ligated inserts destroying the restriction sites for the enzymes (S4A Fig): 5m at 37°C, 5m at 23°C, for 12 cycles (1:2500 dilution of phosphorylated oligo duplex, 2.5ng/μl backbone, 5% v/v DTT, 0.15U/μl BamHI, 0.5U/μl NheI, 1U/μl T4 ligase, 1X restriction buffer; T4 from New England Biolabs, rest from Thermo Fisher Scientific). Plasmids were then amplified in the same manner as the backbone: Plasmids were transformed into DH5a cells, which were then expanded and used for midiprep extraction (Plasmid Midi, Qiagen).

#### miReporter cell line creation

miReporter-590-3p and miReporter-292a-5p plasmids were transfected into ESCs with lipofectamine 3000 (Thermo Fisher Scientific) as per manufacturer’s instruction. Briefly, 125μl DMEM (Sigma-Aldrich) was mixed with 5μl lipofectamine 3000 and vortexed briefly. Separately 125μl DMEM was mixed with 5μl p3000 reagent and 5μg of plasmid and also briefly vortexed. Both mixtures were combined and incubated at room temperature for 5 minutes to create DNA-lipid complexes. 2i medium was removed from pre-seeded ESCs at a confluency of about 70–90% and replaced with serum+LIF medium. DNA-lipid complexes were added to the medium for 24 h. Medium was then aspirated, cells washed twice with PBS, and cells were left to grow for two days in 2i. Transfected cells were selected by hygromycin (100 μg/ml in 2i) for three days. Single clones were selected differently for the two miReporter cell lines. Double-positive, single cells of the miReporter-590-3p cell line were sorted by fluorescence-activated cell sorting in 96-well feeder-coated plates and expanded thereafter. miReporter-292a-5p cells were sparse after passage and single colonies with double-positive cells were picked by hand using a benchtop microscope and a 200μl pipette. Double positive colonies were left to expand on feeders in 48-well plates. Clones were grown for at least two passages to ascertain the stability of the transfection. Reporter activity was confirmed by flow cytometry.

#### miR mimic and inhibitor transfection

Pre-miR miRNA Precursors (Thermo Fisher Scientific) were used as miR mimics. These double stranded RNAs are designed to be processed by the cell to result in mature miRs. miRCURY LNA miRNA Power Inhibitor (Qiagen) was used for miR inhibition. This reagent blocks miRs by complementary binding to the mature miR with high affinity (due to the presence of LNA bases). mESCs and both miReporter cell lines were transfected with miR mimic and inhibitors in identical fashion. See S2 Table for a list of all miR reagents. Cells were seeded in 2i medium 48 h prior to transfection in 12-well plates. Half an hour before transfection, the culture medium was replaced by 500 μl of 2i medium supplemented with 10% FBS to allow the cells to flatten. Lipid complexes (Lipofectamine RNAiMax, Thermo Fisher Scientific) were prepared at the ratios recommended by the manufacturer but siRNA was replaced with either miR mimic or miR inhibitor. miR inhibitor, pre-miR or negative control stock (10 mM), were mixed with 3 μl of Lipofectamine RNAiMax solution and KO DMEM medium to a total volume of 75 μL. We considered 100 nM of mimic/inhibitor in this mixture a 1X concentration. The obtained transfection mix was incubated for 5 min at RT before being added to the cell medium. After 24h (or 48h for the 2X inhibitor experiments on the miReporter cells), the transfected cells were collected and fixed in 4% PFA for 15 min at 4°C. See also section ‘flow cytometry’.

#### RNA and miR sequencing

RNA sequencing libraries were prepared using Illumina’s TruSeq stranded mRNA sample preparation kit. The stranded single end libraries were sequenced on an Illumina HiSeq with 40bp reads and average read depth of 40 million reads per sample. Paired-end libraries for RNA sequencing were sequenced on an Illumina NextSeq 500 with 150bp read length per strand and a read depth of 10 million reads per sample. miRs were extracted from frozen pellets using miRNeasy (Qiagen) kit. Libraries for small RNA sequencing (miR sequencing) were prepared using NEBNext Small RNA Library Prep Set for Illumina (New England Biolabs) and were sequenced on an Illumina NovaSeq 600 with 150bp paired-end reads and read depth between 4 to 15 million per sample. All sequencing data is available through GEO.

#### Mass spectrometry

Pelleted cells were lysed in 400 μl RIPA buffer, except for the sorted cells. Volumes of cell lysate corresponding to 100 μg protein per sample were digested with trypsin using a modified FASP protocol [64]. Subsequently each sample was labeled with TMT 10-plex, 6-plex or 11-plex reagent (Thermo Fisher)) according to the manufacturer’s protocol. All labeled samples were combined into a set-sample. Which labels were assigned to each sample is specified in the specification table. The labeled set-sample was fractionated by electrostatic repulsion-hydrophilic interaction chromatography (ERLIC) run on an HPLC 1200 Agilent system using PolyWAX LP column (200x2.1 mm, 5 μM, 30nm, PolyLC Inc, Columbia, MD) and a fraction collector (Agilent Technologies, Santa Clara, CA). Set-samples were fractionated into a total of 40 ERLIC fractions. Each ERLIC fraction was subsequently further separated by online nano-LC and submitted for tandem mass spectrometry analysis to both LTQ OrbitrapElite or Q exactive high field (HF). One third of each fraction was injected from an auto-sampler into the trapping column (75 um column ID, 5 cm length packed with 5 um beads with 20 nm pores, from Michrom Bioresources, Inc.) and washed for 15 min; the sample was eluted to analytic column with a gradient from 2 to 32% of buffer B (0.1% formic acid in ACN) over 180 min gradient and fed into LTQ OrbitrapElite or Q exactive HF. The instruments were set to run in TOP 20 MS/MS mode method with dynamic exclusion. After MS1 scan in Orbitrap with 60K resolving power, each ion was submitted to an HCD MS/MS with 60K resolving power and to CID MS/MS scan subsequently. All quantification data were derived from HCD spectra.

#### Flow cytometry

Cells were harvested for flow cytometry by washing with PBS and dissociation using Accutase (Sigma-Aldrich). Detached cells were washed and resuspended in 2i. Cells were fixed in 4% paraformaldehyde in medium (Cat. 43368, Alfa Aesar) for 15 min at room temperature. Cells were then centrifuged and the supernatant was removed. In the case of immunostaining, the cells were permeabilized in 1X PBS supplemented with 0.3% Triton X-100 and 1% BSA for 1h at RT and immunostained for CDK7, ACAD8, or EIF4H respectively. Cells were resuspended in 1% BSA (Cat. A2153, Sigma-Aldrich) in PBS and stored at 4°C until the measurement. The fixed cells were measured on a BD LSRFortessa X-20 or LSRII. For the miReporter lines, forward and side scatter were measured as well as Citrine fluorescence (488nm laser, 530/30nm emission filter) and mCherry fluorescence (561nm laser, 610/20 emission filter). For the immunostaining experiments, antibody fluoresccence was using appropriate filter sets.

### Computational methods

#### RNA-seq data pre-processing

Genome assembly mm10 release 93 from Ensembl was used for alignment. First, an RSEM (v1.3.1) reference was created with default settings. Adapter and quality trimming was performed with Trimmomatic (v0.38). Finally all reads were aligned with STAR (v2.6.1a) with the option for stranded libraries enabled. Expected counts from RSEM were used as input for DESeq2 (v1.26) to obtain regularized log_2_ counts with stabilized variance to make comparisons between samples more reliable. From these values regularized counts were obtained and used for all further analyses and as input for batch correction. For the miR transfection samples, genes were required to have expression counts larger than 20. Additionally, sample 3 of the miR-100p-5p mimic was excluded due to low complexity. DESeq2 was used to identify differentially expressed genes and obtain log_2_ fold-changes. For the mimic experiments, 20 genes were differentially expressed between the scrambled control and no treatment control: *Ankmy2*, *B230219D22Rik*, *Col26a1*, *Cyld*, *Elmo3*, *Fam98b*, *Gm9008*, *Med19*, *Mipol1*, *Mmachc*, *Pptc7*, *Psat1*, *Rap1b*, *Rdh13*, *Rfc4*, *Serp1*, *Snx6*, *Srgap3*, *Tjp2* and *Tmem132a (FDR = 0*.*10)*. These 20 genes were excluded from all other comparisons. No genes were differentially expressed for the inhibitor transfections.

#### Combining P-values

To combine p-values we used Fisher’s method which makes use of $T=-2\;\sum_{i=1}^{k}ln(p_{i})∼X_{2k}^{2}$ where *p_i_* are the probabilities, *k* is the number of tests and *T* is chi-square distrubuted with 2*k* degrees of freedom.

#### Proteomics data pre-processing

Peptide search was performed on peptides identified in full RNA-seq data to increase specificity of the protein quantification with MaxQuant. Proteins were quantified from the peptide measurements in the evidence.txt outputs. Reversed peptides and contaminants were removed. Each column in the file was then normalized to the mean. Some peptides for some samples were quantified multiple times, due to multiple mass-spectrometry runs or multiple TMT tags in the same sample mix. These values were averaged. Multiple peptides assigned to a single ensembl gene ID were also averaged to obtain normalized protein expression, which was used for batch correction.

#### RNA-seq and proteomics batch correction

Global expression differences in protein and RNA expression between replicates were corrected using the RemoveBatchEffect function from limma (v3.42.2). The function was applied to the protein and totalRNA datasets separately for three batches: the first replicate, the second replicate and two samples that replaced failed samples of the first replicate. The resulting batch-corrected values were used as input for further analysis.

#### miR-seq data pre-processing

For alignment of miR-seq data we used the same genome release as above with miRnome release 22.1 from miRBase using the mature miR sequences. To prepare the reads we performed adapter and quality trimming with Trimmomatic and obtained a consensus forward sequence using both the forward and reverse read and PEAR (v0.9.6). We next ran bowtie-prepare from bowtie (v1.0.0–1). Finally we quantified each sample with the mapper.pl and quantifier.pl scripts from mirdeep2 (v2.0.1.2). The obtained counts were processed the same way as the RNA-seq data, but separately.

#### Flow cytometry data analysis

Live cell gating and all other analysis of the flow cytometry data was achieved using custom R scripts (FlowCore v1.52.1 [65]). To determine relative down- or upregulation of Citrine expression in the miReporter lines, we calculated the ratio Citrine/mCherry for each cell in the mimic/inhibitor assays and scrambled controls. For the mimic experiments, miReporter downregulation was considered succesfully if the Citrine/mCherry ratio was lower than the 1^st^ percentile of of the signal in miReporter cells transfected with the scrambled control mimic. For the inhibition experiments, miReporter upregulation was considered succesfully if the Citrine/mCherry ratio was higher than the 99^th^ percentile of of the signal in miReporter cells transfected with the scrambled control mimic.

#### Identification of putative miR-mRNA interactions

Putative miR-mRNA interactions were identified with TargetScanMouse release 7.1. The "miR family" table was filtered for expressed miRs and expressed RNAs. Next, all interactions with a cumweightscore lower than -0.3 were filtered out. Finally, to keep only miRs with high dynamics over the time course and high reproducibility the Coefficient of variation (CV) across the mean miR expression of each time point and the mean of the CV’s across the biological replicates was calculated for each miR. A gaussian mixture model was fit to these to values using mclust (v5.4.6), where each distribution has an equal diagonal shape, but with varying volumes ("VEE" modelNames option). Only miR-mRNA interactions from cluster 1 were used because they fit our criteria for high variance and high reproducibility (S1I Fig). The final putative list of miR-mRNA interactions comprised 560 miRs and was used in the miR clustering and model fit (see below).

#### miR clustering

To cluster miRs into sets of similar temporal profiles, miR expression was first averaged per time point. A miR to miR distance matrix was created with 1–Pearson correlation on log_2_-transformed values as the distance. This matrix was then used to perform hierarchical clustering with complete linkage (base R) and the resulting dendrogram was cut into 6 clusters.

#### Calculation of the cytoplasmic fraction (C-fraction)

To obtain a per-gene cytoplasmic fraction a global scaling factor between cytoplasmic and nuclear sequencing reads had to be determined due to input normalization at the library preparation step. For this procedure, genes that had any raw count lower than 10 in any of the samples were removed. Then, the top 500 genes with the lowest variance were fit with the following linear model: $R_{tot}=\beta_{c}\cdot R_{c}+\beta_{n}\cdot R_{n}$, where R_tot_ is total RNA, *R*_*c*_ is cytoplasmic RNA and *R*_*n*_ is nuclear RNA. For each RNA sample regularized log_2_ counts were used. The beta parameters that map cytoplasmic and nuclear values to total RNA values were 0.815 and 0.183 respectively. For each gene *g* the C-fraction was then calculated by:

$$C^{g}=\frac{\beta_{c}\cdot R_{c}^{g}}{\beta_{c}\cdot R_{c}^{g}+\beta_{n}\cdot R_{n}^{g}}$$

#### Rate model fitting

Several rate models were fit for every gene for which data from each measurment modality was available (S1E Fig). RNA and protein expression were scaled by dividing each by the average expression across time. Next a smoothing spline (smooth.spline function, base R) was applied to the total RNA data for each replicate with 7 degrees of freedom (DF). DF was fixed because the automatic inferrence of DF by smooth.spline sometimes sometimes lead to the oversimplification of RNA dynamics and thereby bad fits at the protein level if the protein had more dynamics than the resulting spline. Therefore, a fit was deemed more conservative, if high dynamics were forced at the RNA level at the cost of introducing some noise. smooth.spline was used determine the DF for all other smoothing spline fits using leave-one-out-cross-validation. Smoothing splines fit to the C-fraction were multiplied with the smooth total RNA to get smooth cytoplasmic RNA. miRs that were assigned to each gene were first averaged over replicates and then divided by the miRs maximum value. Smoothing splines were fit to each miR and the smooth miR profiles for each miR cluster were averaged. The differential equations were solved using deSolve (v1.28), given a rate model, parameters, total cytoplasmic RNA and a miR cluster. Instead of using *k*_*s*_ and *k*_*d*_ directly as parameters, log_2_(k_prod_) and log_2_(k_div_), with k_prod_ = k_s_ · k_d_ and k_div_ = k_s_/k_d_, were used as parameters to increase the robustness of the optimization. An additional fit parameter not mentioned in the main text was, P_0_, the protein concentration at t = 0 h, which was used to allow for measurement error on the protein abundance. The parameter *ci* was constrained to be between 0 and the minimal observed protein expression, as co-isolation interference cannot exceed the measured values. α was constrained to be between 0 and 1. Since both α and miR expression scaled to the maximum across time cannot exceed 1, translation can only be completely repressed at peak miR cluster expression. Optimal parameters were found using the optim function (base R). Sum of squared residuals (SSR) were minimized using the "L-BFGS-B" method of the optim function. For the models described by Eqs 2 and 3, there was an initial optimization step minimizing SSR + 10 · log_2_(k_div_) · 2. Due to the scaling of RNA and protein, log_2_(k_div_) is expected to be close to 0 so we penalized any divergence from the expected value to get a better estimate for log_2_(k_prod_) first,. Without the initial regularization, the fits sometimes resulted in extreme parameter values. The resulting parameters were used as initial values for the final unpenalized fits. The Bayesian Information Criterion (BIC) was used to compare models with different numbers of parameters: BIC = k · ln(n)– 2 · SLL, where k is the number of parameters, n is the number of samples, and SLL the sum of log-likelihood. k is 0 for the naive model, 3 for Eqs 1 and 4 for Eqs 2 and 3. n = 8, the number of time points. The error of the fits was assumed to be normally distributed in order to calculate the SLL. When comparing models, the model with the lower BIC was considered superior.

## Supporting information

S1 Fig Quality control of full, cytoplasmic and nuclear RNA-seq; miRNA sequencing and proteomics. (A) Total number of reads for all sequencing samples. (B) Distribution of the number of peptides used for the quantification of each protein. (C) Number of detected genes or miRs in each sample. Individual replicates are plotted as separate bars in (A, C). (D) Distribution of miR-mRNA interactions per gene. (E) Euler diagram of all gene sets. The "miRNA" set indicates genes with predicted miR interaction and the "clean" set is a subset of genes without missing values in either RNA or protein. 53 genes are in the set RNA&Protein&Clean (no miR-mRNA interactions), 13 genes are in the set RNA&Protein (no miR-mRNA interactions, and some genes have missing values). (F) Correlation of temporal mRNA or protein profiles between the two replicates. Shown are distributions of Pearson’s r across all measured mRNAs or proteins. Batch correction improves the correlation between replicates for the mRNAs. (G) Distribution of the mean cytoplasmic fraction (C-fraction) per gene. (H) Coefficient of variation of C-fraction per gene. (I) Gaussian mixture model based clustering of miRs to select a cluster with high reproducibility across replicates and high variance across time (cluster 1), see Methods.
(TIF)

S2 Fig Model performance comparison with Pearson’s r. (A-D) Pearson’s r distribution of various kinetic models. Corresponding R^2^ distributions are shown in Fig 1. (E) R^2^ distributions of the *cytoplasmic RNA* and *ci* model for all genes. The R^2^ distribution of the subset of genes that are best fit by the *ci* model is shown in Fig 1I. (F) Pearson’s r distribution of the *miR* model and the next best model (either *naive*, *total RNA*, *cytoplasmic RNA* or *ci*). Only genes that are best fit by the *miR* model are shown. Corresponding R^2^ distributions are plotted in Fig 2D.
(TIF)

S3 Fig Candidate miR-mRNA interactions for six genes. Example fit of the miR model for genes *Cdk7*, *Pccb*, *Acad8*, *Mfge8*, *Eif4h* and *Srgap2* (rows). First column: expression of the assigned miRs of a single cluster. Colored lines are individual smoothing spline fits. Second column: Cytoplasmic RNA expression and the effective RNA concentration available for translation (see Methods). Solid lines represent smoothing splines. Third/fourth column: cyRNA and miR model fits.
(TIF)

S4 Fig Dose and timing for miR mimic and inhibitor transfection experiments can be obtained using fluorescent reporters of miR activity. (A) miReporter plasmid, inserts and digestion sites (BamHI and NheI). The insert overhangs are compatible with BamHI and NheI, but block redigestion. See Methods for full cloning strategy. (B) Inhibition of the miR-590-3p reporter transcript by the miR-590-3p mimic for seven time points as measured by flow cytometry. The asterisk indicates the optimal transfection timing shown in D (24h). (C) Fluorescence signal of miR-590-3p reporter for miR-590-3p mimic or scrambled control at optimal transfection conditions. Blue line indicates 1st percentile of reporter/normalizer ratio of the scrambled control. (D) Reduction of inhibition of the miR-292a-5p reporter transcript by the miR-292a-5p inhibitor for three time points at three transfection concentrations as measured by flow cytometry. The asterisk indicates the optimal transfection timing shown in E (2days, 2X). (E) Fluorescence signal of miR-292a-5p reporter for miR-292a-5p inhibitor or scrambled control at optimal transfection conditions. Blue line indicates 99th percentile of reporter/normalizer ratio of the scrambled control.
(TIF)

S5 Fig Differential expression of predicted targets after miR mimic and miR inhibitor transfection. (AB) Expression levels (regularized counts scaled to scrambled control) of *Cdk7*, *Leo1*, *Mfge8*, *Pccb*, *Rab8a*, and *Srgap2* after miR mimic (A) and miR inhibitor (B) transfection. P-value shown is for an uncorrected one-sided test (see Methods). Differential expression of two more targets is shown in Fig 4C. Note that *Leo1* was not predicted to be regulated by miR-216a-5p and is included as a negative control.
(TIF)

S6 Fig miR mimic and miR inhibitor versus control fold change distributions. Expression fold changes relative to scrambled control after nine different miR mimic and inhibitor transfections separately for six of our proposed targets. The boxed genes are our proposed targets. Red color indicates significantly differentially expressed genes (P_adj_ < = 0.01). Note that *Leo1* was not predicted to be regulated by miR-216a-5p and is included as a negative control.
(TIF)

S7 Fig miR mimics and inhibitors cause effects in the same direction on ACAD8 and EIF4H protein abundance. Flow cytometry of ACAD8 and EIF4H immunostaining in 3 biological replicates of mESCs treated with miR-433-3p or miR-467e-5p mimics, inhibitors or the respective scrambled controls.
(TIF)

S1 Table List of candidate interactions. (XLSX)

S2 Table List of miR reagents. (XLSX)

S3 Table List of RNA-seq samples. (XLSX)
