# Supplementary figures and images for: Integration of a multi-omics stem cell differentiation dataset using a dynamical model
Source: PLoS Genet. 2023 May 11;19(5):e1010744. doi: 10.1371/journal.pgen.1010744 (PMC10204997; doi:10.1371/journal.pgen.1010744)

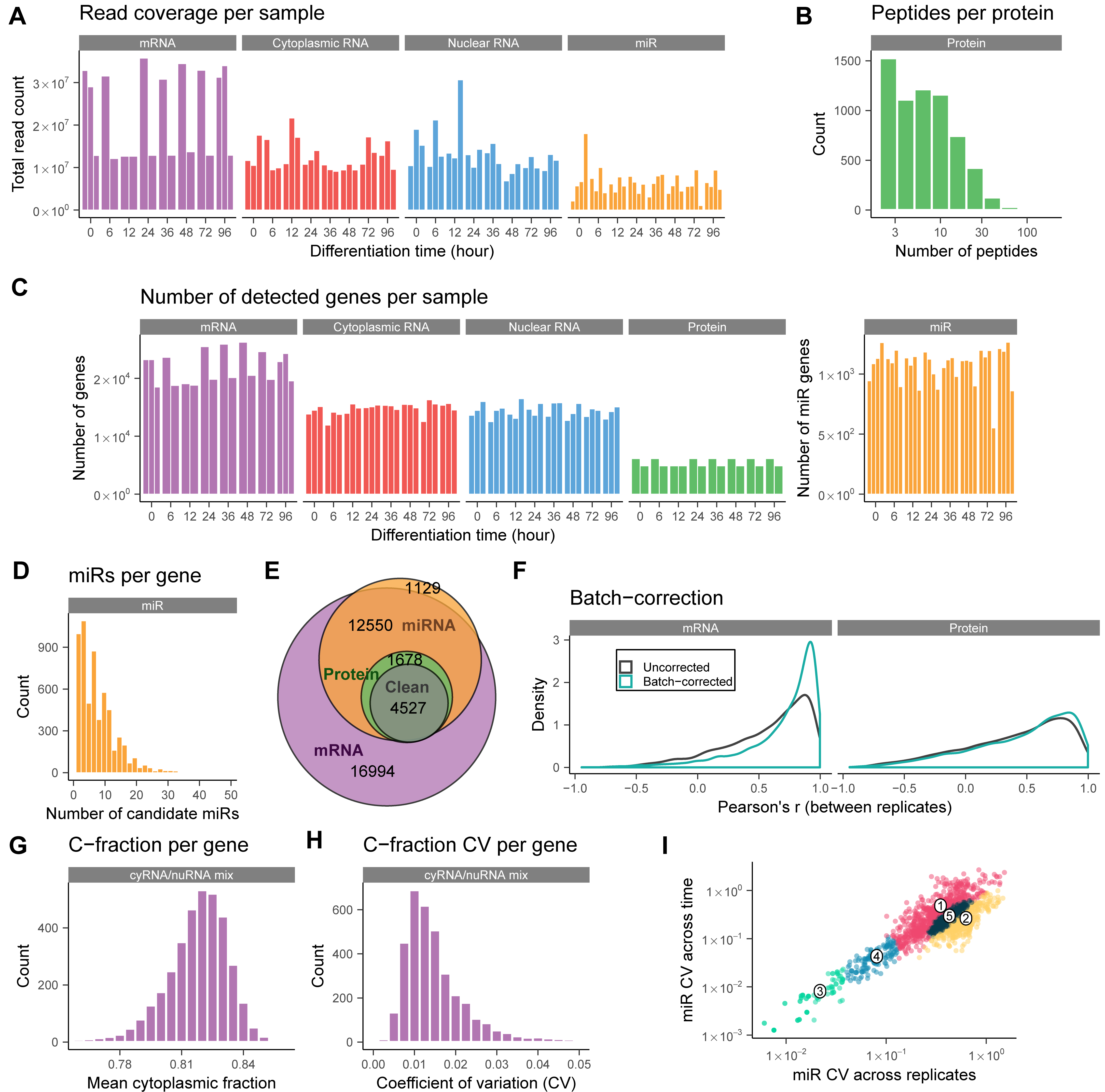

Supplement: S1 Fig — (A) Total number of reads for all sequencing samples. (B) Distribution of the number of peptides used for the quantification of each protein. (C) Number of detected genes or miRs in each sample. Individual replicates are plotted as separate bars in (A, C). (D) Distribution of miR-mRNA interactions per gene. (E) Euler diagram of all gene sets. The "miRNA" set indicates genes with predicted miR interaction and the "clean" set is a subset of genes without missing values in either RNA or protein. 53 genes are in the set RNA&Protein&Clean (no miR-mRNA interactions), 13 genes are in the set RNA&Protein (no miR-mRNA interactions, and some genes have missing values). (F) Correlation of temporal mRNA or protein profiles between the two replicates. Shown are distributions of Pearson’s r across all measured mRNAs or proteins. Batch correction improves the correlation between replicates for the mRNAs. (G) Distribution of the mean cytoplasmic fraction (C-fraction) per gene. (H) Coefficient of variation of C-fraction per gene. (I) Gaussian mixture model based clustering of miRs to select a cluster with high reproducibility across replicates and high variance across time (cluster 1), see Methods. (TIF) [file pgen.1010744.s001.tif]

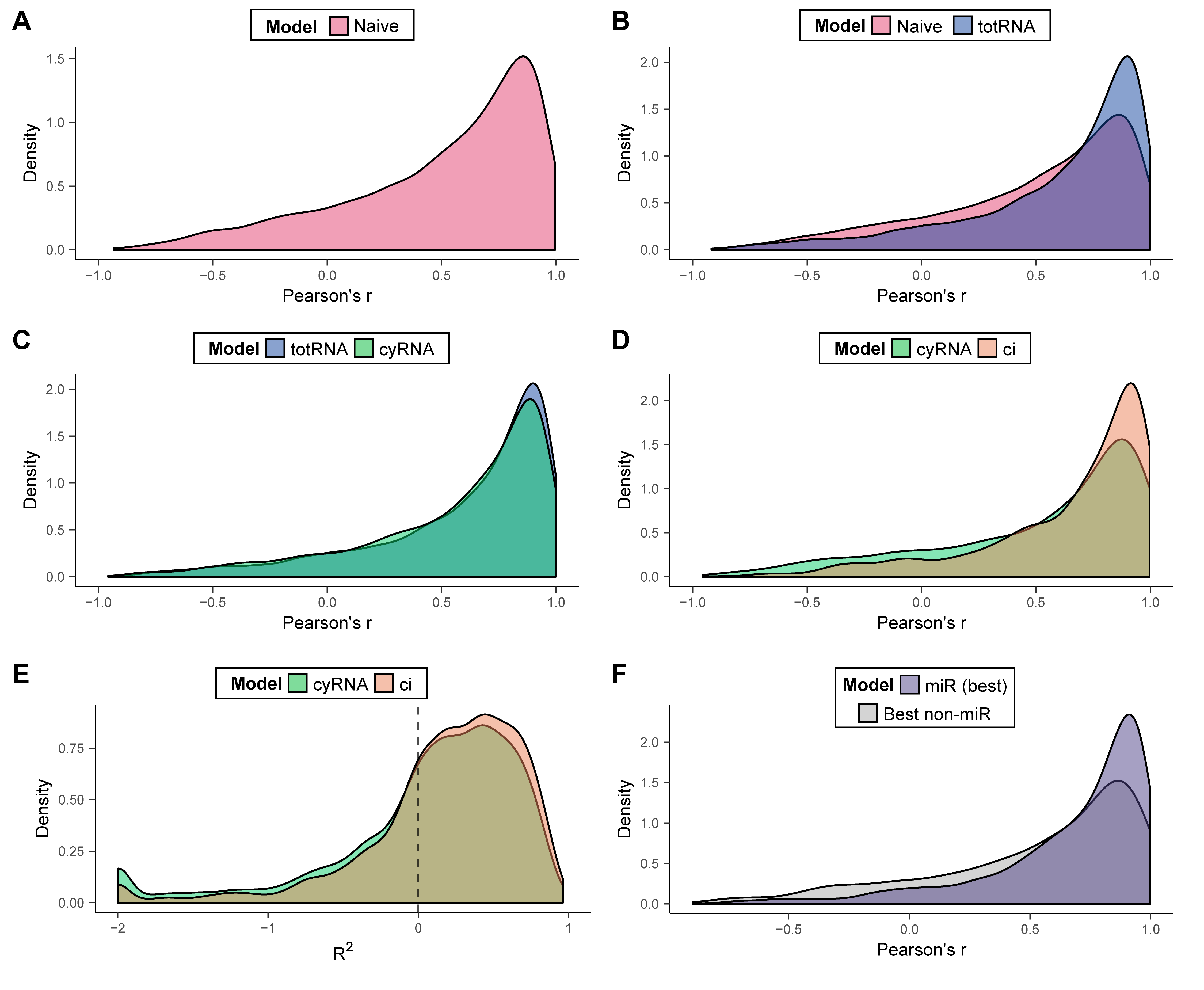

Supplement: S2 Fig — (A-D) Pearson’s r distribution of various kinetic models. Corresponding R2 distributions are shown in Fig 1. (E) R2 distributions of the cytoplasmic RNA and ci model for all genes. The R2 distribution of the subset of genes that are best fit by the ci model is shown in Fig 1I. (F) Pearson’s r distribution of the miR model and the next best model (either naive, total RNA, cytoplasmic RNA or ci). Only genes that are best fit by the miR model are shown. Corresponding R2 distributions are plotted in Fig 2D. (TIF) [file pgen.1010744.s002.tif]

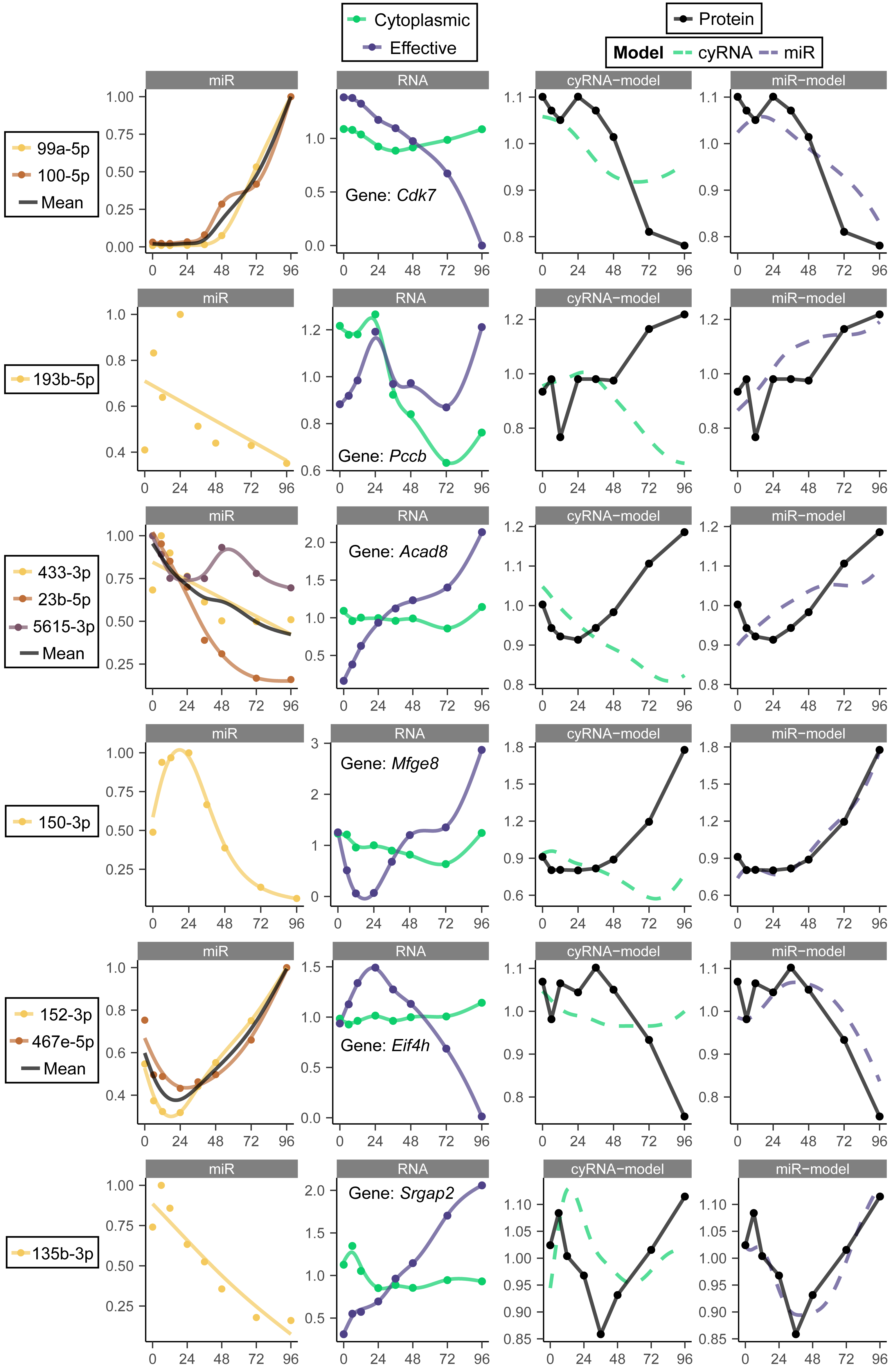

Supplement: S3 Fig — Example fit of the miR model for genes Cdk7, Pccb, Acad8, Mfge8, Eif4h and Srgap2 (rows). First column: expression of the assigned miRs of a single cluster. Colored lines are individual smoothing spline fits. Second column: Cytoplasmic RNA expression and the effective RNA concentration available for translation (see Methods). Solid lines represent smoothing splines. Third/fourth column: cyRNA and miR model fits. (TIF) [file pgen.1010744.s003.tif]

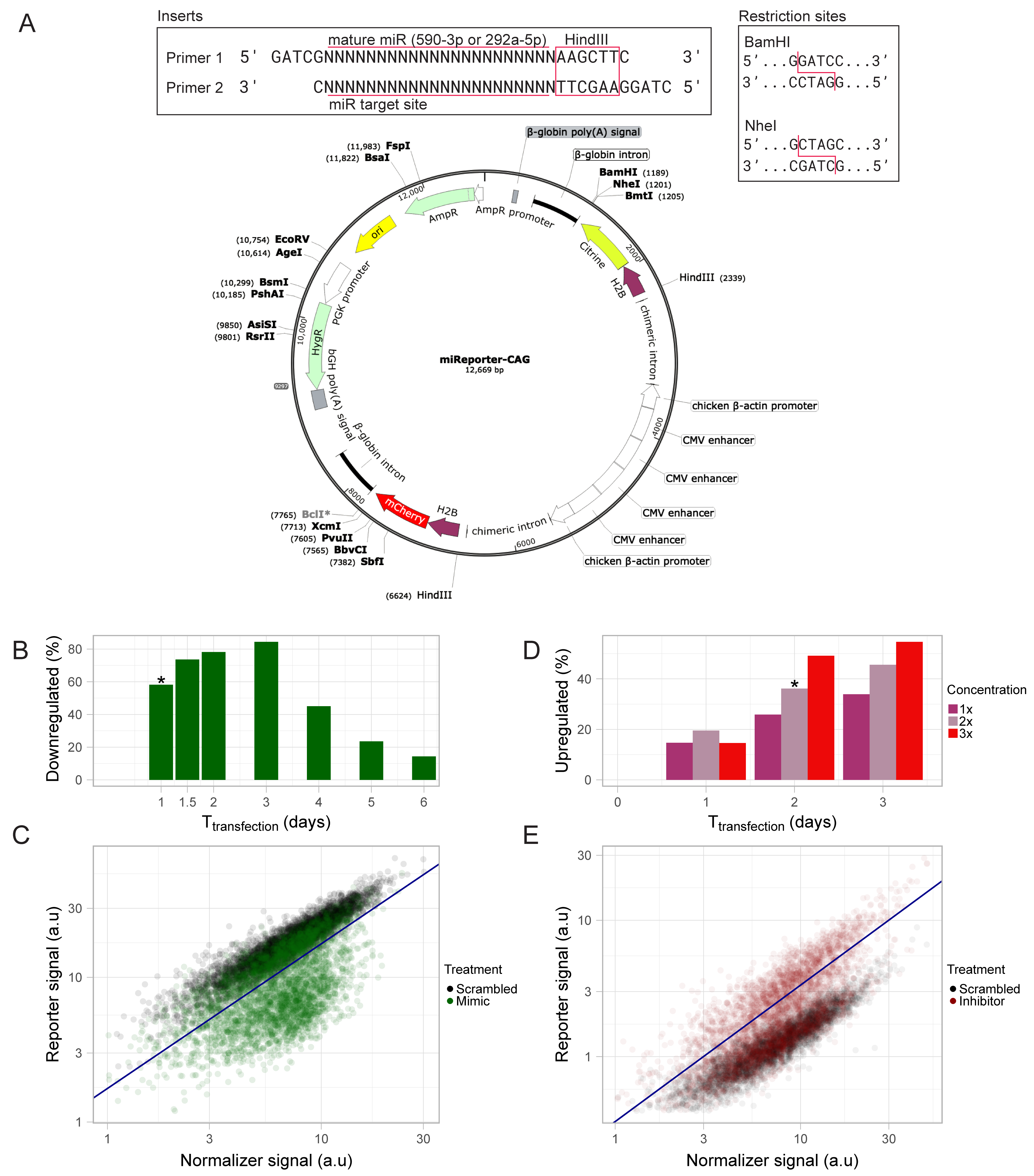

Supplement: S4 Fig — (A) miReporter plasmid, inserts and digestion sites (BamHI and NheI). The insert overhangs are compatible with BamHI and NheI, but block redigestion. See Methods for full cloning strategy. (B) Inhibition of the miR-590-3p reporter transcript by the miR-590-3p mimic for seven time points as measured by flow cytometry. The asterisk indicates the optimal transfection timing shown in D (24h). (C) Fluorescence signal of miR-590-3p reporter for miR-590-3p mimic or scrambled control at optimal transfection conditions. Blue line indicates 1st percentile of reporter/normalizer ratio of the scrambled control. (D) Reduction of inhibition of the miR-292a-5p reporter transcript by the miR-292a-5p inhibitor for three time points at three transfection concentrations as measured by flow cytometry. The asterisk indicates the optimal transfection timing shown in E (2days, 2X). (E) Fluorescence signal of miR-292a-5p reporter for miR-292a-5p inhibitor or scrambled control at optimal transfection conditions. Blue line indicates 99th percentile of reporter/normalizer ratio of the scrambled control. (TIF) [file pgen.1010744.s004.tif]

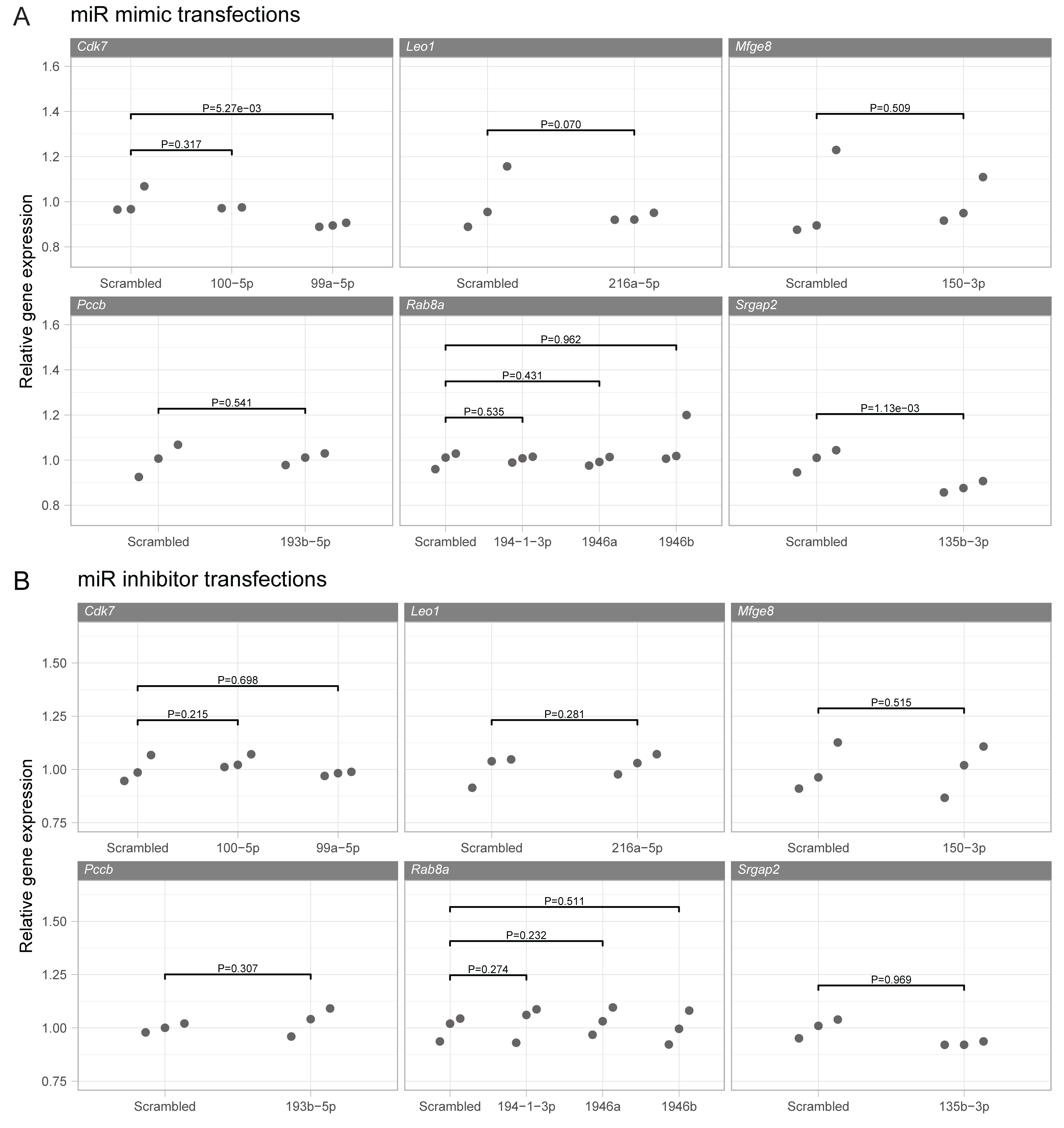

Supplement: S5 Fig — (AB) Expression levels (regularized counts scaled to scrambled control) of Cdk7, Leo1, Mfge8, Pccb, Rab8a, and Srgap2 after miR mimic (A) and miR inhibitor (B) transfection. P-value shown is for an uncorrected one-sided test (see Methods). Differential expression of two more targets is shown in Fig 4C. Note that Leo1 was not predicted to be regulated by miR-216a-5p and is included as a negative control. (TIF) [file pgen.1010744.s005.tif]

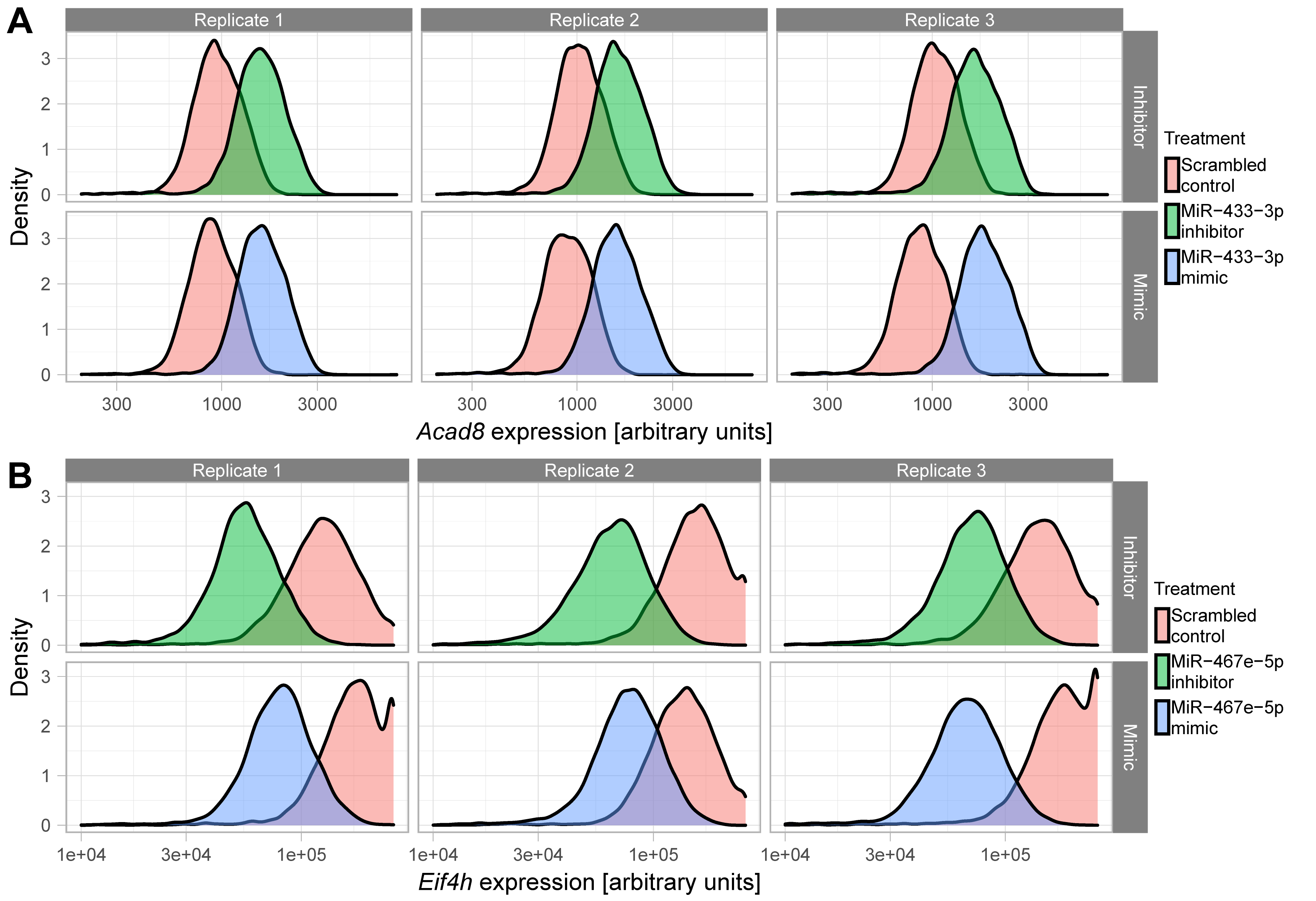

Supplement: S7 Fig — Flow cytometry of ACAD8 and EIF4H immunostaining in 3 biological replicates of mESCs treated with miR-433-3p or miR-467e-5p mimics, inhibitors or the respective scrambled controls. (TIF) [file pgen.1010744.s007.tif]
